# Development and validation of a temporal staging-based nomogram for 1-year mortality in sepsis-associated acute kidney injury

**DOI:** 10.3389/fcimb.2026.1844250

**Published:** 2026-08-06

**Authors:** Jingrong Liu, Kaizhen Han, Hui Pei, Luanluan Zhang, Xianfei Ding, Ke Li, Jiafeng Xie, Yahui Huang, Dong Xu, Zhiqiang Zhu, Tongwen Sun

**Affiliations:** 1 Department of Emergency ICU, The First Affiliated Hospital of Zhengzhou University, Zhengzhou University, Zhengzhou, China; 2 Department of Critical Care Medicine, Department of Emergency Medicine, The First Affiliated Hospital of Zhengzhou University, Henan Engineering Research Center for Critical Care Medicine, Henan Key Laboratory of Critical Care Medicine, Henan Key Laboratory of Sepsis in Health Commission, Zhengzhou Key Laboratory of Sepsis, Henan Sepsis Diagnosis and Treatment Center, Zhengzhou, China

**Keywords:** clinical outcomes, lactate normalization, mortality, prognostic factor, sepsis-associated acute kidney injury, temporal staging

## Abstract

**Background:**

Sepsis-associated acute kidney injury (SA-AKI) is a life-threatening condition with poor prognosis. However, due to significant inter-individual variability in disease progression, clinicians lack reliable tools to stratify patients based on long-term mortality risk.

**Objective:**

This study explores how the timing of SA-AKI (early vs. late onset) affects survival, based on the latest ADQI consensus, and aims to build a simple nomogram to predict 1-year mortality.

**Methods:**

A retrospective cohort study enrolled 422 SA-AKI patients (from 3273 sepsis patients) admitted to the First Affiliated Hospital of Zhengzhou University between January 2020 and December 2024. Patients were stratified into early-onset SA-AKI(E-SA-AKI, within 48 hours of sepsis diagnosis) and late-onset SA-AKI (L-SA-AKI, 48 hours-7 days post-sepsis) groups, with a maximum 5-year follow-up.

**Results:**

The study found that most SA-AKI cases were early-onset (E-SA-AKI vs. L-SA-AKI: 73% vs. 27%). The median age of patients was 65 years, with 72% males. The primary infection sources were abdominal (41.9%) and pulmonary/urinary (36.3%). The cumulative survival rates at 28 days, 90 days, 1 year, and 5 years were 50.5%, 44.8%, 38.9%, and 33.9%, respectively. Both short-term and long-term survival rates were significantly higher in E-SA-AKI patients than L-SA-AKI patients(all *P* < 0.001). Using LASSO and multivariable Cox regression analyses, a prognostic model was developed, identifying five independent predictors of one-year mortality:L-SA-AKI and septic shock as risk factors, while high albumin level, 28-day renal function recovery, and 48-hour lactate normalization as protective factors. The model’s C-statistic (C-index)was 0.79, demonstrating good discrimination and calibration in both the training set [AUC (95% CI): 0.897(0.853–0.931)] and the validation set (AUC(95% CI): 0.909 [0.839–0.951)]. Decision curve analysis indicated favorable clinical utility.

**Conclusion:**

The timing of SA-AKI onset is a critical prognostic stratification indicator. A one-year mortality risk nomogram model constructed based on the temporal staging, septic shock, albumin level, 28-day renal function recovery status, and 48-hour lactate normalization provides important reference for clinical decision-making.

## 1 Introduction

Sepsis-associated acute kidney injury (SA-AKI) is a common and life-threatening complication in the intensive care unit (ICU), occurring in approximately 40%–50% of patients with sepsis and 60%–80% of those with septic shock (Peerapornratana et al., 2019; Bellomo et al., 2017; Donaldson et al., 2024; Takeuchi et al., 2025). SA-AKI significantly increases short-term and long-term mortality, prolongs hospital stays, and imposes a substantial public health burden (Peerapornratana et al., 2019; White et al., 2023). Although the management principles for sepsis and acute kidney injury (AKI) alone have been relatively well-established, precise risk stratification and prognostic prediction tools specifically for the SA-AKI phenotype remain scarce (Fan et al., 2023; Li et al., 2023). Furthermore, existing general critical illness scoring systems, such as the Sequential Organ Failure Assessment (SOFA) or Acute Physiology and Chronic Health Evaluation II (APACHE II), are not designed specifically for SA-AKI, resulting in limited predictive efficacy and a failure to incorporate key dynamic indicators reflecting the unique pathophysiological processes of SA-AKI (Legrand et al., 2024; Wang et al., 2026). Therefore, the development of a prognostic model capable of accurately identifying high-risk patients and guiding individualized clinical decisions is crucial for improving the long-term outcomes of patients with SA-AKI.

The occurrence and progression of SA-AKI is a dynamic process, and the timing of AKI onset may contain important prognostic information. The 28th Acute Disease Quality Initiative (ADQI) Workgroup proposed stratifying SA-AKI into early SA-AKI (E-SA-AKI, AKI occurring within 48 hours of sepsis diagnosis) and late SA-AKI (L-SA-AKI, AKI occurring between 48 hours and 7 days after sepsis diagnosis) (Zarbock et al., 2023). Preliminary evidence suggests that early and late SA-AKI may reflect distinct injury mechanisms (Legrand et al., 2024). However, several critical knowledge gaps remain. The comparative clinical characteristics and prognostic significance of E-SA-AKI and L-SA-AKI under the latest ADQI definition have yet to be systematically validated in real-world cohorts, making it uncertain whether the timing of AKI onset independently predicts short- and long-term mortality beyond established confounders. This knowledge gap is further compounded by the limitations of existing predictive models for SA-AKI, which predominantly rely on static admission parameters and fail to incorporate time-sensitive dynamic indicators — such as lactate clearance and renal function recovery —that reflect the evolving nature of the disease.

To address these gaps, this study utilized the ADQI consensus framework to systematically evaluate the association between SA-AKI onset timing and patient survival. Through a comprehensive analysis of baseline demographics, infection profiles, organ dysfunction severity, and longitudinal response parameters, we aimed to identify independent risk and protective factors for 1−year mortality. Based on these factors, we developed and internally validated a temporal staging−based nomogram for predicting 1−year mortality to provide clinicians with a readily interpretable, bedside−applicable tool for early risk stratification and informed decision−making in patients with SA−AKI.

## 2 Methods

### 2.1 Data source and study population

**Figure 1:**
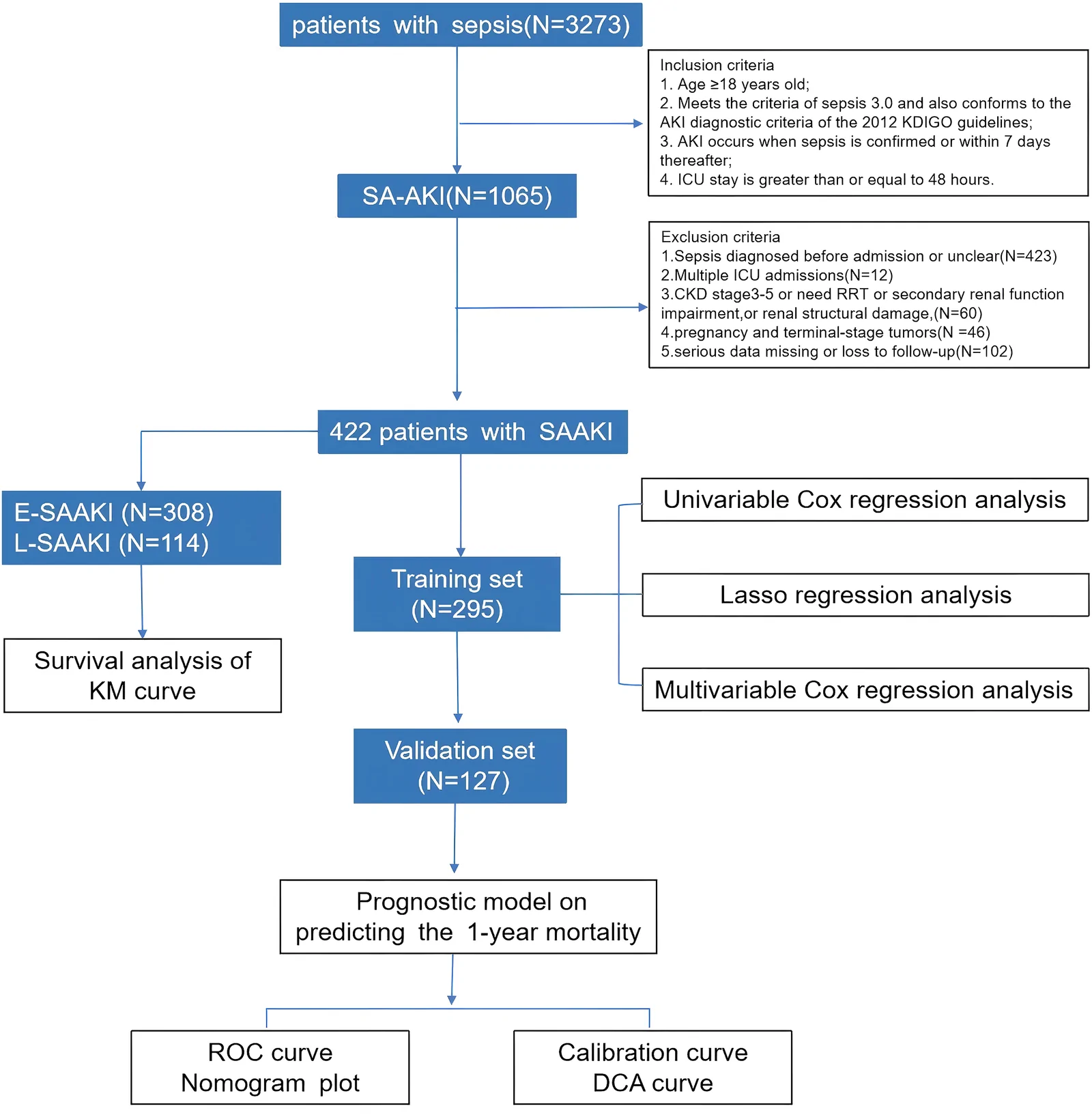
Flowchart of patients enrollment in the study.

Data were obtained from sepsis patients admitted to the intensive care unit (ICU) at the First Affiliated Hospital of Zhengzhou University between January 1, 2019, and December 1, 2024. Inclusion criteria were: (1) Age ≥ 18 years; (2) ICU stay ≥ 48 hours; (3) concurrent diagnosis of sepsis 3.0 and acute kidney injury (AKI)per the Kidney Disease: Improving Global Outcomes (KDIGO) 2012, during ICU admission (Kellum et al., 2013; Singer et al., 2016); (4) AKI onset at the time of sepsis diagnosis or within 7 days thereafter (Zarbock et al., 2023). The baseline serum creatinine level was defined as the lowest value within 3 months before admission; if unavailable, the lowest value during ICU stay was used. All exclusion criteria were established in accordance with the 28th ADQI consensus and previously published retrospective SA-AKI cohort studies, to minimize confounding renal injury factors and ensure homogeneity of the study population (Takeuchi et al., 2025; White et al., 2023; Zarbock et al., 2023; Hong et al., 2026).Exclusion criteria included: (1) sepsis diagnosed before admission or unclear; (2) stage 3 or higher chronic kidney disease or requirement for renal replacement therapy; (3) structural kidney abnormalities, or secondary causes of renal impairment, such as rhabdomyolysis, hepatorenal or cardiorenal syndrome, or nephrotoxic drug–induced injury; (4) pregnancy or advanced-stage malignancy (Nelson-Piercy et al., 2025; Kellum et al., 2021); (5) Multiple admissions to the ICU; (6) severe data loss or loss to follow-up. For patients with multiple ICU admissions during the same hospitalization, only data from the first admission were included. Finally, 422 patients with SA-AKI who met the inclusion criteria were included in our study (**Figure 1**). The study was conducted in accordance with the guidelines of the Helsinki Declaration and approved by the Ethics Committee of the First Affiliated Hospital of Zhengzhou University (2025-KY-0313-001), with waived informed consent for this retrospective analysis. The study was reported in accordance with the STROBE (Strengthening the Reporting of Observational Studies in Epidemiology) reporting guidelines.

### 2.2 Definitions

The diagnosis of sepsis is based on the Sepsis-3 criteria, which requires clear evidence of infection or a high suspicion of infection, along with an increase of ≥2 points in the Sequential Organ Failure Assessment (SOFA) score (Singer et al., 2016). The definition of AKI is based on the KDIGO guidelines, which include an increase in serum creatinine (SCr) of ≥0.3 mg/dL within 48 hours from the baseline value, or a urine output of <0.5 mL·kg^-^¹·h^-^¹ for at least 6 hours (Kellum et al., 2013). These two time thresholds are standardized clinical cutoffs recommended by KDIGO for consistent early AKI screening. The definition of sepsis-associated AKI (SA-AKI) refers to the definition of SA-AKI proposed in the 28th ADQI consensus, which is the coexistence of sepsis and AKI, with AKI occurring within 7 days after the diagnosis of sepsis (Zarbock et al., 2023).

### 2.3 Data collection

Clinical data were retrieved from the electronic medical record system of our hospital. The variable panel was formulated by integrating core static predictors widely adopted in prior SA-AKI cohort studies, with additional time-dependent dynamic indicators and renal recovery prognostic metrics incorporated to address limitations of existing static-only predictive models. Including baseline demographic characteristics (age, sex, height, weight; body mass index (BMI) was calculated), past medical history (hypertension, diabetes, coronary heart disease, chronic cardiac insufficiency, cerebrovascular disease, chronic lung disease, malignancy), Acute Physiology and Chronic Health Evaluation II (APACHE II) score, and Sequential Organ Failure Assessment (SOFA) score.

Additional data collected comprised the source of infection, presence of shock, total duration of vasoactive drug use, body temperature, mean arterial pressure, heart rate, and Glasgow Coma Scale (GCS) score during the first 24 hours after diagnosis. Laboratory parameters from the first 24 hours after diagnosis were also recorded, including arterial blood gas indicators pH, arterial partial pressure of oxygen (PaO_2_), partial pressure of carbon dioxide (PaCO_2_), calculated oxygenation index (PaO_2_/FiO_2_), blood lactate level, base excess, and actual bicarbonate, complete blood count (white blood cell count, neutrophil percentage, hematocrit, platelet count), liver function tests [total bilirubin, alanine aminotransferase (ALT), aspartate aminotransferase (AST), albumin (ALB)], coagulation profile [prothrombin time (PT), activated partial thromboplastin time (APTT), fibrinogen (Fib), fibrin degradation products (FDP), prothrombin activity], inflammatory markers (PCT, IL-6, CRP), and Cardiac markers [Cardiac troponin, N-terminal pro-B-type Natriuretic Peptide (NT-proBNP)].

Furthermore, the total fluid intake (intravenous and oral) and total fluid output (urine output, drainage volume, ultrafiltration volume, and gastrointestinal losses) during the first 24 hours of ICU stay were recorded. Data were also collected on the number of organ failures during the treatment period, the time of renal function recovery and the duration of renal impairment in recovered patients.

Based on the timing of AKI occurrence and following the ADQI consensus definition, patients were categorized into E-SA-AKI (AKI occurring within 48 hours of sepsis diagnosis) and L-SA-AKI (AKI occurring between 48 hours and 7 days after sepsis diagnosis).

### 2.4 Patients follow-up and clinical outcomes

Follow-up procedures and outcome definitions were established based on standardized endpoints used in prior sepsis outcome studies (Rhee et al., 2017; Prescott and Angus, 2018) and followed the STROBE guidelines for observational cohort studies. The last follow-up was performed on December 1, 2025. Overall survival was defined as the time from sepsis diagnosis to all-cause death. The primary outcome measure was one-year all-cause mortality in patients with sepsis. The secondary outcome measures included short-term (28-day and 90-day) and long-term (1-year and 5-year) mortality rates after the diagnosis of sepsis. These timepoints were selected in accordance with conventions established by the Sepsis-3 definitions (Shankar-Hari et al., 2016) and are consistent with large-scale SA-AKI outcome studies (Peerapornratana et al., 2019). Patients who were still alive or lost to follow-up at the time of the last follow-up were censored.

### 2.5 Development and validation of the model

We generated a random number seed and randomly divided the data into a training set and a validation set at a 7:3 ratio for model development and internal validation. In the training set, univariate Cox regression analysis was first performed to screen factors associated with 1-year mortality risk. To prevent overfitting and identify the most essential predictors, we subsequently applied ten-fold cross-validation Least Absolute Shrinkage and Selection Operator (LASSO) regression, which applies an L1 penalty to shrink coefficients of less informative variables to zero. The optimal penalty parameter λ was selected using the λ.1se criterion. Furthermore, a prognostic prediction model was constructed using multivariate Cox regression analysis based on the variables selected by LASSO. The predictive value of the model was evaluated using the area under the receiver operating characteristic (ROC) curve, calibration curves, and decision curves analysis (DCA). A nomogram is further drawn to visualize the model. Logarithmic transformation was applied to skewed predictor variables. The proportional hazards assumption was tested using Schoenfeld residual analysis, with a non-significant result (P > 0.05) indicating the assumption was satisfied. The results were presented with HR (95% CI).

### 2.6 Statistical analysis

Continuous variables were presented as median (interquartile range [IQR]) or mean ± standard deviation (SD). Normally distributed continuous data were compared via Student’s t-test, while non-normally distributed data were analyzed with the non-parametric Mann-Whitney U test. Categorical variables were described using numerical values (percentages). The chi-square test was used for comparisons when all expected cell frequencies were ≥ 5; otherwise, Fisher’s exact test was applied. Survival analysis was conducted using the Kaplan-Meier method, and the log-rank test was used to compare differences between groups. All statistical analyses were performed using IBM SPSS 26.0 and R 4.2.2 software, with a significance level of P<0.05.

## 3 Results

### 3.1 Patient characteristics

As shown in **Table 1**, among the SA-AKI patients, 73% of patients (308/422) developed AKI within 48 hours post-sepsis, which was defined as early-onset SA-AKI (E-SA-AKI). Additionally, 27% (114/422) of the patients developed AKI 2 to 7 days after SA-AKI diagnosis, classified as L-SA-AKI. The median age of SA-AKI patients was 65 years (IQR, 53.0–74.0 years), 72% (304/422) were male, 76.1% (321/422) presented with septic shock, and the primary sources of infection were abdominal (41.9%, 177/422), followed by pulmonary and urinary tract (36.3%, 153/422). During hospitalization, 64.9% (274/422) of the patients experienced two or more organs failure. The highest AKI stage during hospitalization was predominantly AKI stage 3, accounting for 50.0% (211/422).

**Table 1:** Baseline characteristics of patients with SA-AKI.

Variables	Overall (N = 422)	E-SA-AKI (N = 308)	L-SA-AKI (N = 114)
Sex			
Female	118 (28.0%)	88 (28.6%)	30 (26.3%)
Male	304 (72.0%)	220 (71.4%)	84 (73.7%)
**Age, years**	65.0 (53.0–74.0)	65.0 (52.0–74.0)	65.0 (56.0–74.0)
BMI, kg/m^2^			
<18.5	18 (4.3%)	15 (4.9%)	3 (2.6%)
18.5-25.0	239 (56.6%)	169 (54.9%)	70 (61.4%)
≥25.0-28.0	137 (32.5%)	106 (34.4%)	31 (27.2%)
≥28	28 (6.6%)	18 (5.8%)	10 (8.8%)
SOFA score	8 (6–10)	8 (6–10)	8 (5–10)
**APACHEII score**	19 (15–26)	19 (15–25)	20 (15–27)
**GCS score**	13 (11–15)	14 (11–15)	12 (10–15)
Comorbidities			
Hypertension	178 (42.2%)	142 (46.1%)	36 (31.6%)
Diabetes	135 (32.0%)	100 (32.5%)	35 (30.7%)
Coronary heart disease	75 (17.8%)	50 (16.2%)	25 (21.9%)
Chronic heart failure	91 (21.6%)	69 (22.4%)	22 (19.3%)
Cerebrovascular disease	76 (18.0%)	53 (17.2%)	23 (20.2%)
Chronic pulmonary disease	32 (7.6%)	17 (5.5%)	15 (13.2%)
Chronic liver disease	22 (5.2%)	11 (3.6%)	11 (9.6%)
Malignancy	13 (3.1%)	10 (3.2%)	3 (2.6%)
Rheumatoid arthritis	14 (3.3%)	11 (3.6%)	3 (2.6%)
Cardiac arrest	21 (5.0%)	14 (4.5%)	7 (6.1%)
Number of organ failures			
1	49 (11.6%)	37 (12.0%)	12 (10.5%)
2	274 (64.9%)	197 (64.0%)	77 (67.5%)
3	91 (21.6%)	67 (21.8%)	24 (21.1%)
4	8 (1.9%)	7 (2.3%)	1 (0.9%)
New complications			
Myocardial infarction	15 (3.6%)	11 (3.6%)	4 (3.5%)
Acute heart failure	86 (20.4%)	63 (20.5%)	23 (20.2%)
Septic shock	321 (76.1%)	230 (74.7%)	91 (79.8%)
Site of infection			
Abdominal	177 (41.9%)	143 (46.4%)	34 (29.8%)
Non-abdominal (lung, urinary)	153 (36.3%)	103 (33.4%)	50 (43.9%)
Other (intracranial, bloodstream, skin/soft tissue)	92 (21.8%)	62 (20.1%)	30 (26.3%)
AKI stage			
1	75 (17.8%)	55 (17.9%)	20 (17.5%)
2	136 (32.2%)	94 (30.5%)	42 (36.8%)
3	211 (50.0%)	159 (51.6%)	52 (45.6%)
Physiological parameters			
MAP, mmHg	82.2(66.1–96.0)	80.8 (64.0–96.0)	89.7 (72.1–97.8)
Heart rate, bpm	106 ± 24.8	107 ± 24.2	101 ± 25.9
Respiratory rate, breaths/min	23 (19–29)	23 (20–30)	23 (19–28)
Temperature, °C	37.2 (36.8–38.2)	37.2 (36.7–38.0)	37.5 (36.8–38.4)
First 24-hour data			
Urine output, mL	1420 (753–2300)	1325 (590–2070)	1705 (1200–2695)
Fluid balance			
< 0 mL	109 (25.8%)	69 (22.4%)	40 (35.1%)
> 0 mL	313 (74.2%)	239 (77.6%)	74 (64.9%)
**Vasoactive agent duration, days**	2.4(1.0–4.0)	2.4 (0.0–4.0)	2.7 (1.0–4.8)
Laboratory parameters			
Calculated oxygenation index (PaO_2_/FiO_2_)			
< 200	173 (41.0%)	119 (38.6%)	54 (47.4%)
≥ 200	249 (59.0%)	189 (61.4%)	60 (52.6%)
PH			
<7.35	129 (30.6%)	107 (34.7%)	22 (19.3%)
≥7.35	293 (69.4%)	201 (65.3%)	92 (80.7%)
PaCO_2_, mmHg	30 (25.7–36.1)	29.0 (25.0–35.0)	31.6 (27.1–38.4)
HCO_3_^-^, mmol/L	20.1 (16.4–23.7)	18.95 (15.8–22.6)	22.55 (19.4–25.2)
Lactate, mmol/L	2.3 (1.4–3.6)	2.4 (1.4–3.7)	2 (1.4–2.8)
Base excess, mmol/L	-5.1 (-9.3–0.9)	-6.1 (-10.3--2.0)	-2.7 (-6.2-1.0)
WBC, 10^9^/L			
≤9.5	147 (34.8%)	103 (33.4%)	44 (38.6%)
>9.5	275 (65.2%)	205 (66.6%)	70 (61.4%)
Platelet count, 10^9^/L	126 (70.3–200.0)	126.5 (70.0–204.8)	118.5 (71.5–182.5)
Neutrophil percentage, %	89.8 (83.9–93.9)	90.3 (84.8–94.2)	88.05 (81.7–92.8)
Hematocrit			
<0.30	103 (24.4%)	67 (21.8%)	36 (31.6%)
0.30-0.46	283 (67.1%)	212 (68.8%)	71 (62.3%)
≥0.46	36 (8.5%)	29 (9.4%)	7 (6.1%)
Inflammatory markers			
PIV	0.24 (0.12–0.40)	0.24 (0.12–0.40)	0.24 (0.13–0.39)
Procalcitonin, ng/mL	20.2 (4.6–69.2)	25.8 (8.4–84.0)	8.7 (1.8–21.8)
Interleukin-6, pg/mL	193.7 (63.8–1916.5)	234.5 (62.9–2029.0)	155.3 (64.9–633.4)
C-reactive protein, mg/L	156.9 (74.9–262.5)	171.2 (85.8–270.5)	126.0 (56.3–259.9)
Electrolytes and Renal function			
Potassium, mmol/L	4.0 (3.5–4.5)	4.0 (3.5–4.5)	3.9 (3.5–4.4)
Sodium, mmol/L	137.5 (133.0–142.0)	137.0 (133.0–142.0)	138.0 (134.5–143.0)
Urea/Creatinine ratio	42.1 (33.3–55.2)	40.6 (32.2–51.4)	47.6 (36.4–59.5)
eGFR, mL/min/1.73m²	35.2 (22.3–59.3)	31.2 (20.7–43.8)	62.9 (34.8–86.7)
Liver function and Lipids			
Total bilirubin, μmol/L	17.9 (10.7–36.0)	18.8 (10.6–37.9)	16.4 (10.8–27.3)
Direct bilirubin, μmol/L	10.5 (5.9–24.0)	11.3 (6.2–28.0)	8.6 (5.5–15.7)
ALT, U/L	28.0 (15.0–66.8)	28.0 (15.0–82.5)	28.0 (15.3–52.0)
AST, U/L	39.0 (24.0–109.8)	41.5 (25.0–123.5)	36.0 (22.3–80.8)
Albumin, g/L	28.8 (25.3–33.0)	28.7 (25.3–32.9)	29.1 (25.3–33.3)
Total cholesterol, mmol/L	2.6 (2.1–3.4)	2.6 (2.1–3.4)	2.7 (2.1–3.5)
Triglycerides, mmol/L	1.5 (0.9–2.2)	1.5 (0.9–2.2)	1.4 (1.0–2.2)
Cardiac markers			
Cardiac troponin, ng/L	0.05(0.02–0.19)	0.06 (0.02–0.20)	0.042(0.01–0.16)
NT-proBNP, pg/mL	3240.0 (1014.8–8746.5)	3889.0 (1359.5–9184.8)	1664.5 (418.5–6043.5)
Coagulation profile			
Fibrinogen, g/L	4.2 (3.0–5.3)	4.3 (3.2–5.3)	4.0 (2.7–5.6)
FDP, mg/L	16.6 (9.1–35.9)	17.5 (9.7–39.9)	13.4 (8.2–31.4)
D-dimer, mg/L FEU	3.0 (1.2–7.3)	3.2 (1.2–7.9)	2.6 (1.2–5.9)
INR	1.2 (1.1–1.5)	1.3 (1.1–1.6)	1.2 (1.1–1.4)
PT, s	13.8 (12.2–16.5)	14.1 (12.3–17.3)	13.1 (12.1–14.9)
APTT, s	32.6 (28.4–37.4)	32.6 (28.4–37.6)	32.7 (28.5–36.4)
Prothrombin activity, %	72 (55.85–88)	70 (53–86)	78.4 (63–90)
Outcomes			
Renal function recovery at 28 days	197 (46.7%)	157 (51.0%)	40 (35.1%)
Lactate normalization within 48 hours	236 (55.9%)	183 (59.4%)	53 (46.5%)
RRT during hospitalization	165 (39.1%)	120 (39.0%)	45 (39.5%)
Tracheal intubation	144 (34.1%)	106 (34.4%)	38 (33.3%)

SA-AKI, Sepsis-Associated Acute Kidney Injury; E-SA-AKI, Early SA-AKI; L-SA-AKI, Late SA-AKI; BMI, Body Mass Index; SOFA, Sequential Organ Failure Assessment; APACHE II, Acute Physiology and Chronic Health Evaluation II; GCS, Glasgow Coma Scale; AKI, Acute Kidney Injury; MAP, Mean Arterial Pressure; PaO_2_/FiO_2_, Ratio of arterial oxygen partial pressure to fractional inspired oxygen; WBC, White Blood Cell count; PIV, Pan-Immune-Inflammation Value; eGFR, estimated Glomerular Filtration Rate; ALT, Alanine Aminotransferase; AST, Aspartate Aminotransferase; NT-proBNP, N-terminal pro-B-type Natriuretic Peptide; FDP, Fibrin Degradation Products; INR, International Normalized Ratio; PT, Prothrombin Time; APTT, Activated Partial Thromboplastin Time; RRT, Renal Replacement Therapy. Data are presented as n (%), median (IQR), or mean ± SD.

The overall survival rates for all patients at 28 days, 90 days, 1 year, and 5 years were 50.5% (213/422), 44.8% (189/422), 38.9% (164/422), and 33.9% (143/422), respectively. The rates of major adverse kidney events (MAKE) at 28 days and 90 days were 61.4% (259/422) and 58.8% (248/422), respectively.

Moreover, 28-day (55.8% vs. 36.0%, P<0.05) and 90-day (49.7% vs. 31.6%, P<0.05) short-term survival rates, as well as 1-year (44.2% vs. 24.6%, P<0.05) and 5-year (38.6% vs. 21.1%, P<0.05) long-term survival rates, were significantly higher in E-SA-AKI patients than in L-SA-AKI patients. L-SA-AKI patients had a significantly higher incidence of 28-day (75.4% vs. 56.2%, P<0.05) and 90-day (71.9% vs. 53.9%, P<0.05) MAKE than E-SA-AKI patients (**Supplementary Table 1**).

**Figure 2:**
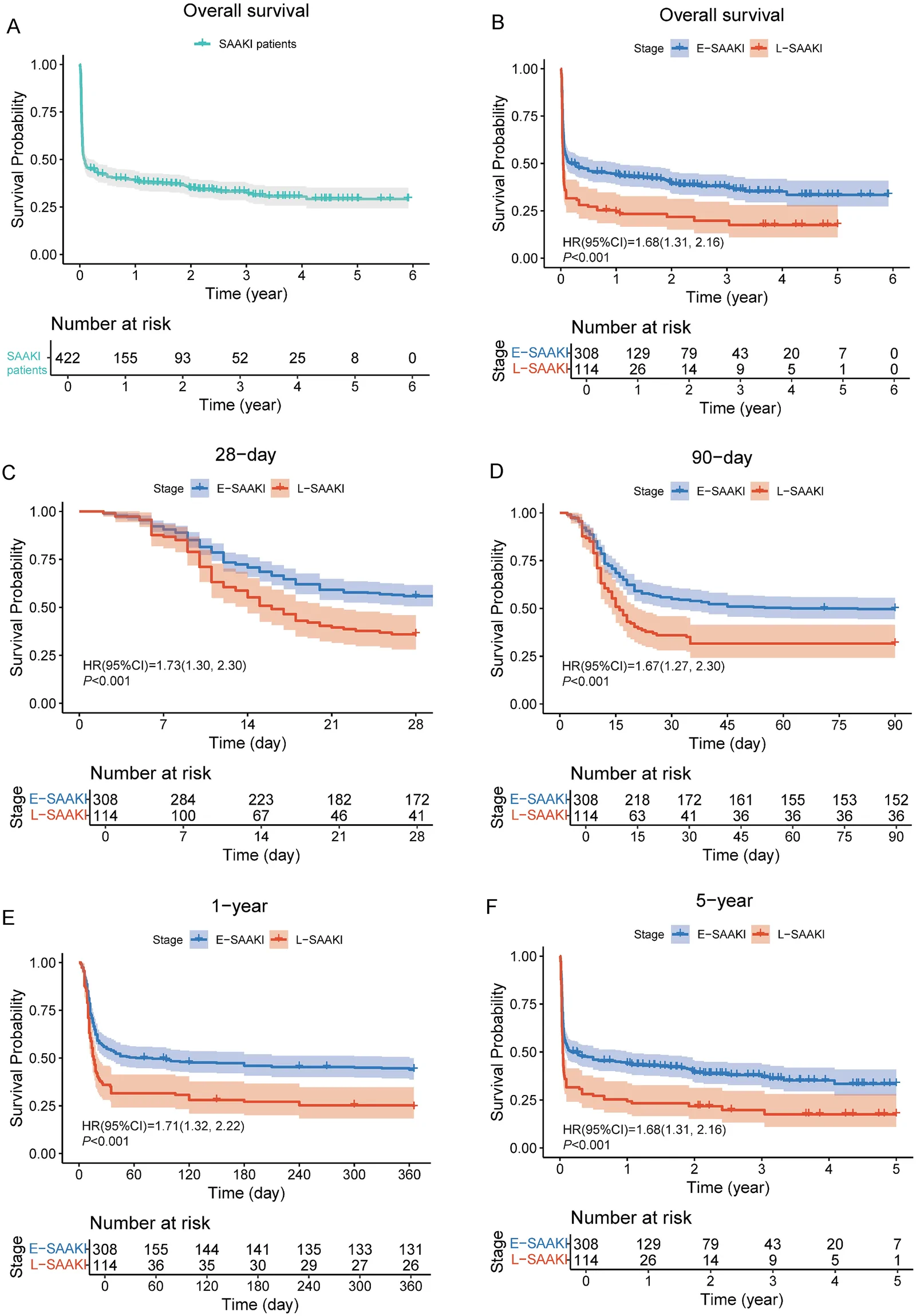
Kaplan–Meier survival curves of patients with SA-AKI. **(A)** Overall survival for all SA-AKI patients. **(B–F)** Comparisons of overall survival, 28-day survival, 90-day survival, 1-year survival, and 5-year survival between the E-SA-AKI (blue line) and L-SA-AKI (red line) groups.

Furthermore, Kaplan-Meier survival analyses demonstrated that E-SA-AKI patients had higher overall survival, 28 days, 90 days, 1 year, and 5 years survival rates than those in patients with L-SA-AKI (P<0.001) (**Figure 2**).

A total of 295 patients (69.9%) were assigned to the training set, while the remaining 127 patients (30.1%) comprised the internal validation set. The characteristics of SA-AKI patients in the training and validation datasets are presented in **Supplementary Table 2**. There were no significant differences between two sets on demographic, clinical, and outcome variables, including SA-AKI stage, AKI severity, APACHE II score, and the rate of renal recovery at 28 days, confirming the suitability of the split for validation purposes.

### 3.2 Model development and validation

Univariate Cox regression identified 17 factors significantly associated with 1-year mortality in SA-AKI patients. Among these, L-SA-AKI, male sex, low BMI, higher APACHE II score, cardiac arrest, septic shock, higher AKI stage, receipt of renal replacement therapy (RRT) during hospitalization, elevated PaCO_2_, and elevated potassium were all risk factors for 1-year mortality (P<0.05). Conversely, renal function recovery within 28 days, lactate normalization within 48 hours, higher urine output in the first 24 hours, higher calculated oxygenation index, elevated pH, higher ALB levels, and elevated Fib were protective factors (P<0.05) (**Supplementary Table 3**). Furthermore, we performed LASSO regression analysis on these 17 factors associated with 1-year mortality in the derivation dataset. This method aimed to prevent overfitting and identify the most essential predictive variables. The results showed that, under the penalty parameter λ.se, the number of variables with non-zero coefficients was six: stage (L-SA-AKI), APACHE II score, ALB, septic shock, renal function recovery within 28 days, and lactate normalization within 48 hours (**Supplementary Figure 2**). In multivariate Cox regression analysis, L-SA-AKI [HR (95% CI): 1.40 (1.03–1.91), P = 0.031], ALB [HR (95% CI): 0.15 (0.07–0.30), P<0.001], renal function recovery within 28 days [HR (95% CI): 0.24 (0.17–0.34), P<0.001], lactate normalization within 48 hours [HR (95% CI): 0.39 (0.28–0.54), P<0.001], and septic shock [HR (95% CI): 2.24 (1.43–3.52), P<0.001] were independent predictors of 1-year mortality (**Table 2**). Prognostic equation: 0.34 × L-SA-AKI (1=yes, 0=no) - 1.90 × ln(ALB, g/L) - 1.44 × 28-day renal function recovery (1=yes, 0=no) - 0.94 × 48-hour lactate normalization (1=yes, 0=no) + 0.81 × septic shock (1=yes, 0=no). Moreover, higher ALB levels, renal function recovery within 28 days, and lactate normalization within 48 hours are independent protective factors, while L-SA-AKI and septic shock are independent risk factors for 1-year mortality of patients with SA-AKI.

**Table 2:** Univariate and multivariate Cox regression analysis of variables associated with the 1-year mortality of SA-AKI patients.

Variables	Univariable COX analysis		Multivariate COX analysis	
	HR (95%CI)	P value	HR (95%CI)	P value
**Stage(L-SA-AKI)**	1.71 (1.26–2.32)	**0.001**	1.40(1.03–1.91)	**0.031**
**Sex (Male)**	1.51 (1.07–2.13)	**0.019**		
BMI, kg/m^2^				
<18.5	2.07 (1.08–3.97)	**0.028**		
≥25.0-28.0	0.80 (0.58–1.11)	0.177		
≥28	0.88 (0.47–1.64)	0.694		
**APACHEII score**	1.07 (1.05–1.09)	**<0.001**		
Cardiac arrest	3.45 (1.94–6.14)	**<0.001**		
Septic shock	2.94 (1.90–4.56)	**<0.001**	2.24(1.43–3.52)	**<0.001**
AKI stage				
2 vs 1	1.38 (0.84–2.26)	0.199		
3 vs 1	2.47 (1.57–3.87)	**<0.001**		
Renal function recovery at 28 days	0.18 (0.13–0.26)	**<0.001**	0.24(0.17–0.34)	**<0.001**
Lactate normalization within 48h	0.27 (0.20–0.37)	**<0.001**	0.39(0.28–0.54)	**<0.001**
RRT during hospitalization	2.09 (1.56–2.80)	**<0.001**		
**First 24-hour u**rine output (**increasing 500 ml/24 h)**	0.86 (0.80–0.92)	**<0.001**		
Calculated oxygenation index (PaO_2_/FiO_2_)**(≥200)**	0.62 (0.46–0.82)	**0.001**		
**PaCO2**	1.02 (1.01–1.03)	**0.004**		
**PH(≥7.35)**	0.55 (0.41–0.73)	**<0.001**		
**ln(**Potassium)	2.38 (1.13–5.00)	**0.022**		
**ln(**Albumin)	0.25 (0.13–0.46)	**<0.001**	0.15(0.07–0.30)	**<0.001**
**ln(**Fibrinogen)	0.61 (0.47–0.80)	**<0.001**		

SA-AKI, Sepsis-Associated Acute Kidney Injury; L-SA-AKI, Late-onset Sepsis-Associated Acute Kidney Injury; HR (95% CI), Hazard Ratio (95% Confidence Interval); BMI, Body Mass Index; APACHE II, Acute Physiology and Chronic Health Evaluation II; AKI, Acute Kidney Injury; RRT, Renal Replacement Therapy; PaO_2_, Partial Pressure of Arterial Oxygen; FiO_2_, Fraction of Inspired Oxygen; PaCO_2_, Partial Pressure of Arterial Carbon Dioxide; pH, Potential of Hydrogen. Bold P values indicate statistical significance (P < 0.05).

**Figure 3:**
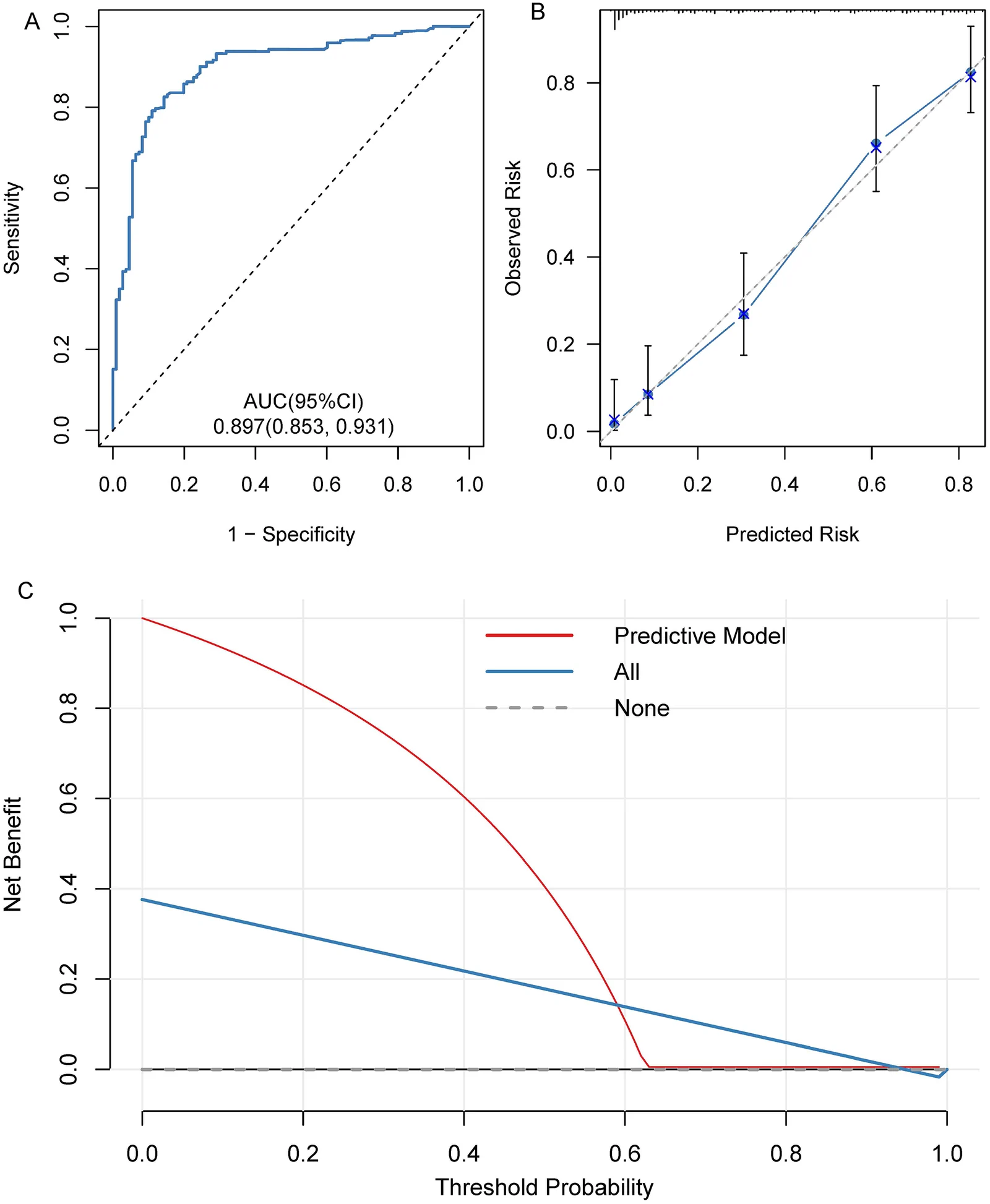
The predictive value of prognostic models on predicting the 1-year mortality of patients with SA-AKI in the training set. **(A)** ROC curve with AUC of 0.897 (95% CI: 0.853–0.931). **(B)** Calibration curve. **(C)** Decision curve analysis.

**Figure 4:**
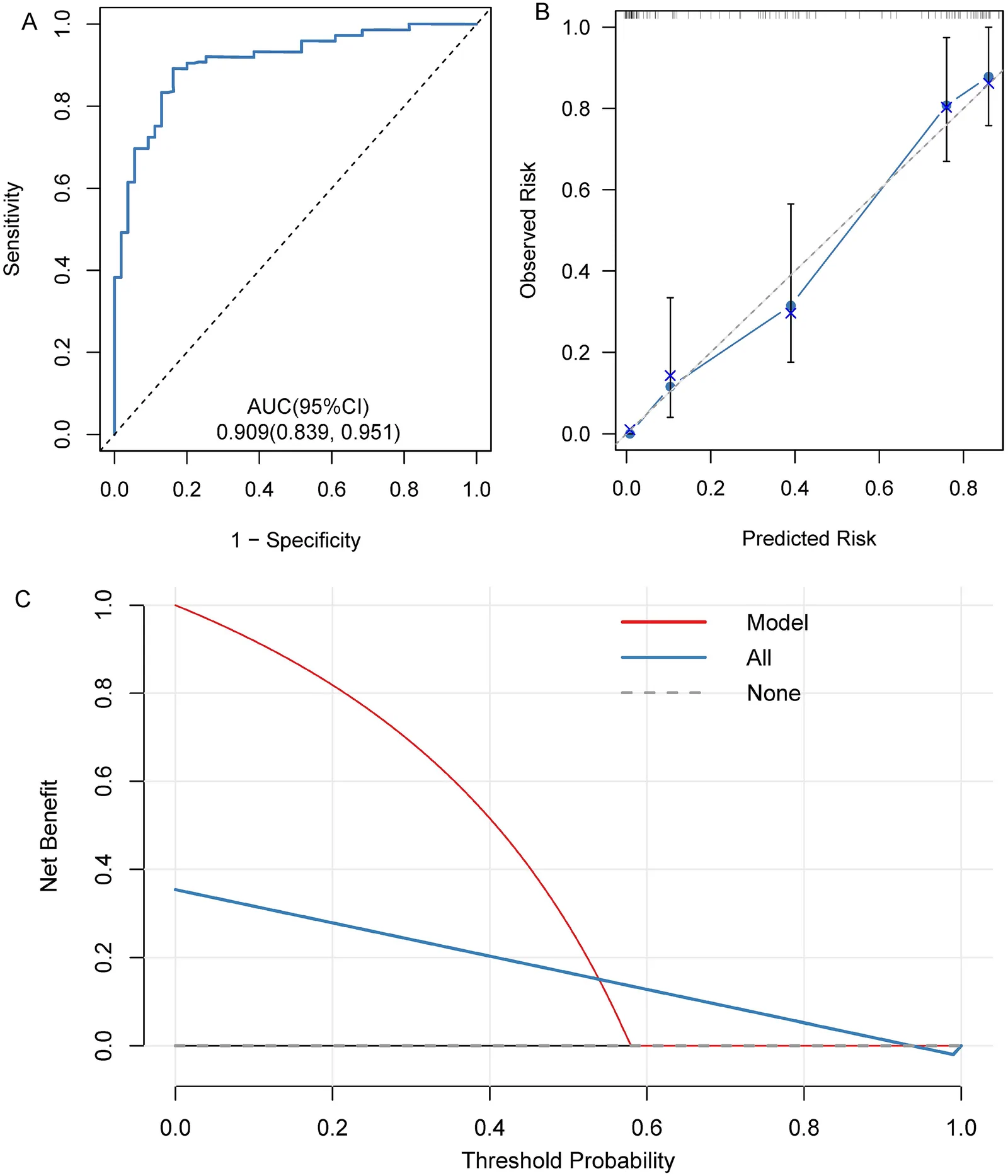
The predictive value of prognostic models on predicting the 1-year mortality of patients with SA-AKI in the validation set. **(A)** ROC curve with AUC of 0.909 (95% CI: 0.839–0.951). **(B)** Calibration curve. **(C)** Decision curve analysis.

**Figure 5:**
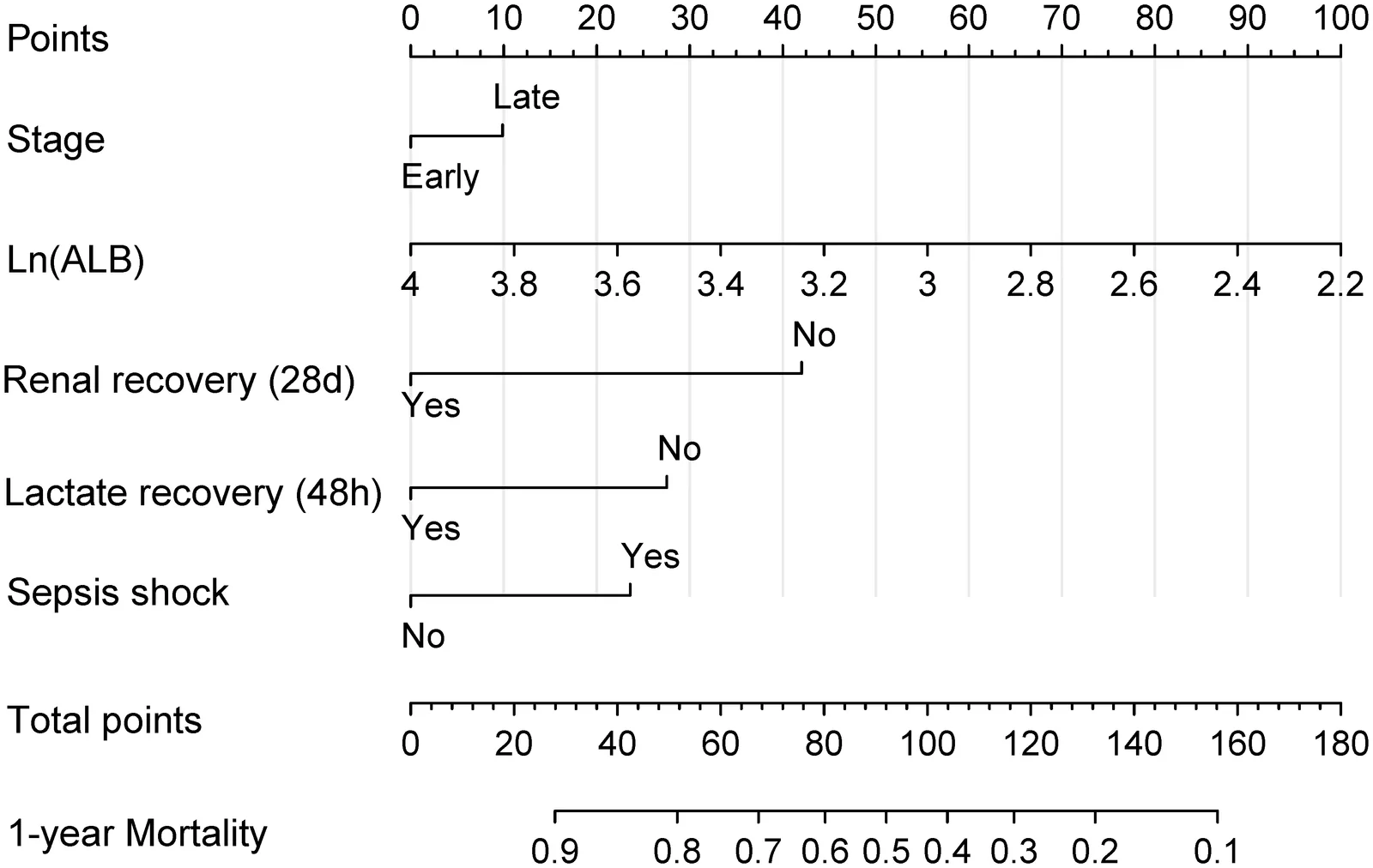
Nomogram plot of prognostic model on predicting the 1-year mortality of patients with SA-AKI.

The prognostic model constructed based on these five factors had a C-index of 0.79.The likelihood ratio test indicated that the model as a whole was significant (P<0.05) and satisfied the proportional hazards assumption (P>0.05). In training set, the AUC for predicting 1-year mortality was 0.897 (95% CI: 0.853–0.931). The calibration curve demonstrated high consistency between predicted and actual risk. Decision curve analysis demonstrated that, within a threshold probability range of 0 to 0.6, the red model line remained consistently above both the blue ‘treat none’ line (horizontal at net benefit ≈ 0) and the gray ‘treat all’ line, indicating that the nomogram provides higher net benefit than uniform treatment strategies across this clinically relevant range (**Figure 3**). The net benefit was especially pronounced in the 0.2–0.5 threshold range, where clinical decision uncertainty is greatest. The validation set confirmed a comparable pattern (**Figure 4**), demonstrating the robustness of the model’s clinical utility.” (**Figures 3**, **4**). Furthermore, we developed a nomogram to visualize the 1-year mortality risk for individual patients, facilitating clinical decision-making and revealed that patient with L-SA-AKI, low albumin levels, no renal function recovery within 28 days, lactate non-normalization within 48 hours, and septic shock would have a total score >140 points, corresponding to a predicted 1-year mortality risk of >80%. Conversely, a patient with E-SA-AKI, high albumin levels, renal recovery within 28 days, lactate normalization within 48 hours, and no septic shock would have a total score near zero, corresponding to a very low mortality risk (<10%) (**Figure 5**).

## 4 Discussion

This study was conducted based on the updated definition and temporal stratification criteria for SA-AKI proposed in the latest ADQI consensus (Zarbock et al., 2023). For the first time, we systematically incorporated the onset timing of SA-AKI (i.e., early-onset SA-AKI and late-onset SA-AKI) as a key predictive variable into long-term prognostic modeling. Furthermore, we integrated multidimensional clinical indicators reflecting baseline characteristics, disease severity, organ function recovery, and metabolic status for comprehensive analysis. Ultimately, we identified independent risk and protective factors associated with 1-year all-cause mortality among patients with SA-AKI, and established a well-performing nomogram prediction model for convenient clinical application.

The temporal staging framework proposed by the ADQI consensus has attracted growing interest as an approach to disentangle the pathophysiological heterogeneity of SA-AKI (Zarbock et al., 2023; Legrand et al., 2024). Our finding that E-SA-AKI accounted for approximately 73% of cases aligns with previous reports by Takeuchi et al. (2025), confirming that early-onset AKI predominates the SA-AKI landscape. Although the proportions of E- and L-SA-AKI vary across studies owing to differences in patient populations, the marked predominance of early-onset SA-AKI remains a consistent clinical observation (Kyo et al., 2026; Monard et al., 2024). Furthermore, our study found significant differences in clinical features between early and late SA-AKI, further explaining the reasons for prognostic divergence. Regarding infection focus, abdominal infection was the predominant source in the E-SA-AKI group(accounting for 46.4%), whereas non-abdominal infections (pulmonary and urinary tract infections) accounted for a higher proportion in the L-SA-AKI group(accounting for 43.9%). This pattern raises the possibility that the site of infection may influence the trajectory of renal injury. It is possible that abdominal infections often accompanied by early, rapid and severe inflammatory responses predisposing to rapid kidney injury (Peerapornratana et al., 2019), whereas urinary tract infections have been specifically associated with a higher risk of persistent acute kidney disease (AKD), supporting a link between infection source and sustained renal dysfunction (Peerapornratana et al., 2020). In terms of renal function trajectory, E-SA-AKI patients had significantly higher renal function recovery rates, while L-SA-AKI patients had higher rates of 28-day MAKE (71.9% vs. 53.9%) and in-hospital RRT use (39.5% vs. 39.0%), supporting the ADQI consensus conclusion that renal injury in late SA-AKI is more irreversible.

These distinct clinical profiles were paralleled by marked differences in prognosis between the two phenotypes. E-SA-AKI patients had significantly higher short- and long-term survival than L-SA-AKI patients (all P<0.001), with L-SA-AKI exhibiting nearly half the 1-year survival rate (24.6% vs. 44.2%). Shi et al. (2025) reported similar trends from MIMIC-IV but focused on short-term outcomes without a dedicated prediction model incorporating temporal stage. Our study advances this by demonstrating that L-SA-AKI retains independent prognostic significance after rigorous multivariable adjustment (HR = 1.40, 95% CI = 1.03-1.91, P = 0.031), consistent with the ADQI consensus on pathophysiological heterogeneity.

The mechanistic underpinnings of the E-SA-AKI versus L-SA-AKI divergence warrant careful consideration. Early-onset SA-AKI may be primarily mediated by sepsis-induced acute inflammation, renal hemodynamic dysfunction, and endothelial injury (Peerapornratana et al., 2019; Menon et al., 2026; Bellomo et al., 2017), and is characterized by reversible pathological changes, as evidenced by the higher 28-day recovery rate in E-SA-AKI patients (51.0%) than in L-SA-AKI patients (35.1%). Thus, timely intervention may facilitate renal function recovery in this subset. In contrast, late-onset SA-AKI may be mostly associated with persistent inflammatory and immune responses, mitochondrial dysfunction and cell cycle arrest/senescence, maladaptive repair of tubular epithelial cells, and activation of pro-fibrotic signaling pathways (TGF-β/SMAD, Wnt/β-catenin), ultimately driving progressive renal parenchymal injury and fibrotic remodeling, a trajectory consistent with the concept of sepsis-associated acute kidney disease (AKD) (Bellomo et al., 2017; Peerapornratana et al., 2020; Kounatidis et al., 2024; Nian et al., 2025; Ozrazgat-Baslanti et al., 2022).

Beyond temporal staging, the four supplementary predictors form a biologically consistent, clinically practical risk system. This five-variable panel offers a new perspective on SA-AKI heterogeneity and facilitates precise intervention. High albumin level was an independent protective factor in this study, a finding consistent with previous research conclusions that “low albumin levels are associated with poor prognosis in SA-AKI” (Liu et al., 2026; Ni and Qin, 2023). Hypoalbuminemia is often accompanied by severe inflammation and capillary leakage, while maintaining higher albumin (ALB) levels can improve vascular permeability, reduce tissue edema, and indirectly protect renal function (Vincent et al., 2016). Lactate normalization within 48 hours was another independent protective factor, reflecting the restoration of tissue perfusion and oxygen metabolism. Delayed lactate clearance is a key manifestation of abnormal metabolic reprogramming in late SA-AKI, and early correction of shock and improvement of tissue hypoxia are crucial for protecting renal function (Legrand et al., 2024; Plata-Menchaca et al., 2026).The 28th ADQI consensus regards renal recovery trajectory as a core metric for stratifying long-term SA-AKI prognosis (Zarbock et al., 2023). This viewpoint is further supported by Menon et al. (2026), who emphasized that AKI duration and recovery patterns are key predictors of lasting clinical outcomes. Consistent with these consensus statements, our multivariate Cox analysis identified 28-day renal function recovery as an independent protective factor against 1-year all-cause mortality (HR = 0.24, P < 0.001), offering quantitative real-world evidence to validate this prognostic framework. Mechanistically, failure to restore renal function within 28 days indicates defective tubular self-repair and irreversible renal parenchymal damage, which represents a pivotal transition point for progression from acute kidney injury to chronic kidney disease (Li et al., 2026). Septic shock, identified as the strongest risk factor (HR = 2.24, 95% CI 1.43–3.52, P < 0.001), arises primarily from tissue hypoperfusion and multiple organ dysfunction induced by severe infection. It forms a vicious cycle with AKI: shock aggravates renal ischemia and hypoxia, whereas renal impairment further exacerbates systemic homeostatic disturbances (Chen et al., 2026; Dunay Silva et al., 2026). In this study, 76.1% of SA-AKI patients developed septic shock, consistent with the findings of White et al (White et al., 2023). Shock as an independent risk factor for SA-AKI prognosis is a core marker of SA-AKI severity. This result emphasizes the critical role of early and aggressive shock correction and improvement of tissue perfusion in improving prognosis.

Currently published SA-AKI prognostic prediction models, both domestically and internationally, mostly focus on in-hospital mortality or short-term prognosis, and concentrate on baseline disease severity scores (such as APACHE II, SOFA) or single biomarkers. There is insufficient exploration of the independent value of the timing of AKI onset—a dynamic, early-available clinical piece of information—in long-term prognosis, and conclusions are inconsistent (Wang et al., 2026; Peerapornratana et al., 2020; Shi et al., 2025; White et al., 2023). The nomogram model constructed in this study has direct application value and guiding significance for clinical practice. The model exhibits excellent performance (C-index=0.79, training set AUC = 0.897). Decision curve analysis confirmed that the model provides net clinical benefit within a threshold probability range of 0 to 0.6 in both the training and validation sets (**Figures 3**, **4**), confirming its clinical applicability across the spectrum from conservative monitoring to aggressive intervention. It can optimize clinical decision-making through risk stratification, avoiding overtreatment in low-risk patients and ensuring focused intervention for high-risk patients. Specifically, for the extremely high-risk subgroup predicted by the model to have a >80% 1-year mortality risk (characterized by L-SA-AKI, low albumin, non-recovery of renal function, non-normalization of lactate, and accompanied by shock), existing conventional treatment strategies may be insufficient. This points towards target populations for future clinical trials designed for targeted intensive therapies (such as earlier renal replacement therapy intervention, biologics targeting specific inflammatory pathways, or aggressive nutritional and metabolic support). Conversely, for patients predicted to have a very low risk (<10%), clinicians may consider adopting more conservative monitoring strategies to rationally allocate intensive care resources. Furthermore, this model can assist in early risk communication and prognosis assessment upon patient admission, providing a quantitative basis for individualized treatment and follow-up plans, potentially improving the overall management of SA-AKI and patient long-term survival.

This study has several limitations. First, as a single-center retrospective observational study, inherent selection bias and unmeasured confounders (e.g., pathogen characteristics, genetic susceptibility, and subtle treatment variations) may persist despite rigorous statistical adjustment, potentially weakening causal inference. Second, the modest sample size and absence of external multi-center validation limit the generalizability of the model. Furthermore, baseline renal function estimated by the lowest in-hospital creatinine level may not precisely reflect pre-admission renal status, and some time-dependent variables were not fully captured. Future prospective multi-center studies are warranted to validate and refine this predictive tool.

## 5 Conclusions

This study confirms that the temporal stratification of SA-AKI(E-SA-AKI vs. L-SA-AKI) is a core determinant of clinical heterogeneity and long-term prognosis, with late-onset SA-AKI patients exhibiting significantly worse short- and long-term prognoses. The prediction model constructed based on the temporal staging, septic shock, ALB level, 28-day renal function recovery status, and 48-hour lactate normalization demonstrates good discriminative ability and clinical utility, enabling precise risk stratification for SA-AKI patients. Based on the latest ADQI consensus, this study provides temporal evidence for research on SA-AKI heterogeneity, offers important references for clinical individualized treatment and guideline updates, and holds significant translational medical potential.

## Data Availability

The original contributions presented in the study are included in the article/**Supplementary Material**. Further inquiries can be directed to the corresponding author.
