# Supplementary material for: Development and validation of a temporal staging-based nomogram for 1-year mortality in sepsis-associated acute kidney injury
Source: Front Cell Infect Microbiol. 2026 Aug 6;16:1844250. doi: 10.3389/fcimb.2026.1844250 (PMC13490152; doi:10.3389/fcimb.2026.1844250)
Supplement: Supplementary file 1 [file DataSheet1.docx]

**Title: Development and Validation of a Temporal Staging-Based Nomogram for 1-Year Mortality in Sepsis-Associated Acute Kidney Injury**

**Running Title:** SA-AKI Prognostic Prediction Model

Jingrong Liu^1✝^, Kaizhen Han^1✝^,Hui Pei^1^,Luanluan Zhang^1^,Xianfei Ding^2^,Ke Li^1^,Jiafeng Xie^1^,Yahui Huang^1^,Dong Xu^1^,Zhiqiang Zhu^1*^,Tongwen Sun^2*^

^1^Department of Emergency ICU, The First Affiliated Hospital of Zhengzhou University, Zhengzhou University, Zhengzhou 450052, China;

^2^Department of Critical Care Medicine, Department of Emergency Medicine, The First Affiliated Hospital of Zhengzhou University, Henan Engineering Research Center for Critical Care Medicine, Henan Key Laboratory of Critical Care Medicine, Henan Key Laboratory of Sepsis in Health Commission, Zhengzhou Key Laboratory of Sepsis, Henan Sepsis Diagnosis and Treatment Center, Zhengzhou 450052, China

^✝^These authors contributed equally to this work and share first authorship.

**^*^Co-corresponding authors：**

^*^Tongwen Sun, MD, PhD,Department of Critical Care Medicine, Department of Emergency Medicine,The First Affiliated Hospital of Zhengzhou University,Zhengzhou University,Jianshedong Road 1, Zhengzhou,450052, China;

Email:suntongwen@163.com.

^*^Zhiqiang Zhu,MD,PhD,Department of Emergency ICU, The First Affiliated Hospital of Zhengzhou University, Zhengzhou University, Jianshedong Road 1,Zhengzhou,450052, China;

Email:[13623810089@126.com](mailto:13623810089@126.com).

**Supplemental Files**

Table of contents

Supplemental Table 1	-	Page 2

Supplemental Table 2	-	Page 3-5

Supplemental Table 3	-	Page 6-8

Supplemental Figure 1	-	Page 9

Supplemental Figure 2	-	Page 9

**Supplemental Table 1**. The impact of early and late stages on prognostic indicators for

patients with AKI

| **Variables** | **Overall**  **(N=422)** | **E-SA-AKI**  **(N=308)** | **L-SA-AKI**  **(N=114)** | **c^2^/Z** | **P value** |
| --- | --- | --- | --- | --- | --- |
| **Status** |  |  |  | 11.483 | 0.001 |
| Survival | 143 (33.9) | 119 (38.6) | 24 (21.1) |  |  |
| Death | 279 (66.1) | 189 (61.4) | 90 (78.9) |  |  |
| **28-days survival** |  |  |  | 13.154 | <0.001 |
| Yes | 213 (50.5) | 172 (55.8) | 41 (36.0) |  |  |
| No | 209 (49.5) | 136 (44.2) | 73 (64.0) |  |  |
| **90-days survival** |  |  |  | 11.019 | 0.001 |
| Yes | 189 (44.8) | 153 (49.7) | 36 (31.6) |  |  |
| No | 233 (55.2) | 155 (50.3) | 78 (68.4) |  |  |
| **1-year survival** |  |  |  | 13.445 | <0.001 |
| Yes | 164 (38.9) | 136 (44.2) | 28 (24.6) |  |  |
| No | 258 (61.1) | 172 (55.8) | 86 (75.4) |  |  |
| **5-year survival** |  |  |  | 11.483 | 0.001 |
| Yes | 143 (33.9) | 119 (38.6) | 24 (21.1) |  |  |
| No | 279 (66.1) | 189 (61.4) | 90 (78.9) |  |  |
| **In-hospital death** |  |  |  | 14.040 | <0.001 |
| No | 336 (79.6) | 259 (84.1) | 77 (67.5) |  |  |
| Yes | 86 (20.4) | 49 (15.9) | 37 (32.5) |  |  |
| **28-days MAKE** |  |  |  | 13.033 | <0.001 |
| No | 163 (38.6) | 135 (43.8) | 28 (24.6) |  |  |
| Yes | 259 (61.4) | 173 (56.2) | 86 (75.4) |  |  |
| **90-days MAKE** |  |  |  | 11.167 | 0.001 |
| No | 174 (41.2) | 142 (46.1) | 32 (28.1) |  |  |
| Yes | 248 (58.8) | 166 (53.9) | 82 (71.9) |  |  |
| **Early onset of partial recovery in renal function** |  |  |  | 10.817 | 0.001 |
| No | 211 (50.0) | 139 (45.1) | 72 (63.2) |  |  |
| Yes | 211 (50.0) | 169 (54.9) | 42 (36.8) |  |  |
| **Time in ICU /d[M (P25, P75)]** | 9(6-12) | 9(6-12) | 10(7-12) | -2.389 | 0.017 |

Abbreviations: E-SA-AKI: Early Sepsis-Associated Acute Kidney Injury; L-SA-AKI: Late Sepsis-Associated Acute Kidney Injury; MAKE: Major Adverse Kidney Events; ICU: Intensive Care Unit; IQR: Interquartile Range.

**Supplemental Table 2**. Clinical characteristics in training set and validation set

| **Variables** | **Overall**  **(N=422)** | **Training set (N=295)** | **Validation set (N=127)** | **P value** |
| --- | --- | --- | --- | --- |
| **Stage** |  |  |  | 0.755 |
| Early | 308(73.0%) | 214(72.5%) | 94(74.0%) |  |
| Late | 114(27.0%) | 81(27.5%) | 33(26.0%) |  |
| **Sex** |  |  |  | 0.725 |
| Female | 118 (28.0%) | 81 (27.5%) | 37 (29.1%) |  |
| Male | 304 (72.0%) | 214 (72.5%) | 90 (70.9%) |  |
| **Age, years** | 65.0 (53.0–74.0) | 66 (53.5–74.0) | 65.0 (52.0–73.0) | 0.445 |
| **BMI, kg/m^2^** |  |  |  | 0.828 |
| <18.5 | 18 (4.3%) | 11 (3.7%) | 7 (5.5%) |  |
| 18.5-25.0 | 239 (56.6%) | 166 (56.3%) | 73 (57.5%) |  |
| ≥25.0-28.0 | 137 (32.5%) | 98 (33.2%) | 39 (30.7%) |  |
| ≥28 | 28 (6.6%) | 20 (6.8%) | 8 (6.3%) |  |
| **SOFA score** | 8 (6–10) | 8 (6–11) | 8 (6–10) | 0.027 |
| **APACHEII score** | 19 (15–26) | 20 (15–26) | 19 (15–25) | 0.907 |
| **GCS score** | 13 (11–15) | 13 (11–15) | 14 (9–15) | 0.341 |
| **Comorbidities** |  |  |  |  |
| Hypertension | 178 (42.2%) | 126 (42.7%) | 52 (40.9%) | 0.736 |
| Diabetes | 135 (32.0%) | 99 (33.6%) | 36 (28.3%) | 0.292 |
| Coronary heart disease | 75 (17.8%) | 57 (19.3%) | 18 (14.2%) | 0.204 |
| Chronic heart failure | 91 (21.6%) | 63 (21.4%) | 28 (22.0%) | 0.874 |
| Cerebrovascular disease | 76 (18.0%) | 60 (20.3%) | 16 (12.6%) | 0.058 |
| Chronic pulmonary disease | 32 (7.6%) | 21 (7.1%) | 11 (8.7%) | 0.583 |
| Chronic liver disease | 22 (5.2%) | 15 (5.1%) | 7 (5.5%) | 0.856 |
| Malignancy | 13 (3.1%) | 9 (3.1%) | 4 (3.1%) | >0.999 |
| Rheumatoid arthritis | 14 (3.3%) | 12 (4.1%) | 2 (1.6%) | 0.310 |
| Cardiac arrest | 21 (5.0%) | 13 (4.4%) | 8 (6.3%) | 0.412 |
| **Number of organ failures** |  |  |  | 0.405 |
| 1 | 49 (11.6%) | 33 (11.2%) | 16 (12.6%) |  |
| 2 | 274 (64.9%) | 186 (63.1%) | 88 (69.3%) |  |
| 3 | 91 (21.6%) | 70 (23.7%) | 21 (16.5%) |  |
| 4 | 8 (1.9%) | 6 (2.0%) | 2 (1.6%) |  |
| **New complications** |  |  |  |  |
| Myocardial infarction | 15 (3.6%) | 11 (3.7%) | 4 (3.1%) | 0.993 |
| Acute heart failure | 86 (20.4%) | 62 (21.0%) | 24 (18.9%) | 0.620 |
| **Septic shock** | 321 (76.1%) | 231 (78.3%) | 90 (70.9%) | 0.100 |
| **Site of infection** |  |  |  | 0.838 |
| Abdominal | 177 (41.9%) | 125 (42.4%) | 52 (40.9%) |  |
| Non-abdominal (lung, urinary) | 153 (36.3%) | 108 (36.6%) | 45 (35.4%) |  |
| Other (intracranial, bloodstream, skin/soft tissue) | 92 (21.8%) | 62 (21.0%) | 30 (23.6%) |  |
| **AKI stage** |  |  |  | 0.904 |
| 1 | 75 (17.8%) | 54 (18.3%) | 21 (16.5%) |  |
| 2 | 136 (32.2%) | 94 (31.9%) | 42 (33.1%) |  |
| 3 | 211 (50.0%) | 147 (49.8%) | 64 (50.4%) |  |
| **Physiological parameters** |  |  |  |  |
| MAP, mmHg | 82.2 (66.1–96.0) | 80.7 (65.2–94.2) | 84.3 (69.5–100.8) | 0.062 |
| Heart rate, bpm | 106 ± 24.8 | 106 ± 23.4 | 105 ± 28.0 | 0.653 |
| Respiratory rate, breaths/min | 23 (20–29) | 23 (19–29) | 23 (20–29) | 0.742 |
| Temperature, °C | 37.2 (36.8–38.2) | 37.2 (36.8–38.0) | 37.3 (36.7–38.5) | 0.777 |
| **First 24-hour data** |  |  |  |  |
| Urine output, ml | 1420 (753–2300) | 1400 (700–2201) | 1500 (828–2500) | 0.509 |
| Fluid balance |  |  |  | 0.309 |
| <0 mL | 109 (25.8%) | 72 (24.4%) | 37 (29.1%) |  |
| >0 mL | 313 (74.2%) | 223 (75.6%) | 90 (70.9%) |  |
| **Vasoactive agent duration, days** | 2.4 (1.0–4.0) | 2.0 (1.0–3.0) | 3.0 (0.0–5.5) | 0.147 |
| **Laboratory parameters** |  |  |  |  |
| Calculated oxygenation index (PaO₂/FiO₂) |  |  |  | 0.082 |
| <200 | 173 (41.0%) | 129 (43.7%) | 44 (34.6%) |  |
| ≥200 | 249 (59.0%) | 166 (56.3%) | 83 (65.4%) |  |
| PH |  |  |  | 0.675 |
| <7.35 | 129 (30.6%) | 92 (31.2%) | 37 (29.1%) |  |
| ≥7.35 | 293 (69.4%) | 203 (68.8%) | 90 (70.9%) |  |
| PaCO₂, mmHg | 30.0 (25.7–36.1) | 29.8 (25.1–36.0) | 30.5 (26.0–37.3) | 0.262 |
| HCO₃⁻, mmol/L | 20.1 (16.4–23.7) | 19.7 (16–23.1) | 21.3 (17.35–24.8) | 0.019 |
| Lactate, mmol/L | 2.3 (1.4–3.6) | 2.3 (1.5–3.6) | 2.1 (1.2–3.6) | 0.093 |
| Base excess, mmol/L | -5.1 (-9.3–-0.9) | -6 (-9.6–-1.4) | -4.2 (-7.4–0.9) | 0.012 |
| WBC, 10⁹/L |  |  |  | 0.618 |
| ≤9.5 | 147 (34.8%) | 105 (35.6%) | 42 (33.1%) |  |
| >9.5 | 275 (65.2%) | 190 (64.4%) | 85 (66.9%) |  |
| Platelet count, 10⁹/L | 126.0 (70.3–200.0) | 127.0 (78.5–203.0) | 115 (63.0–195.5) | 0.149 |
| Neutrophil percentage, % | 89.8 (83.9–93.9) | 90.3 (85–94.3) | 88.5 (82.1–92.9) | 0.005 |
| Hematocrit |  |  |  | 0.018 |
| <0.30 | 103 (24.4%) | 83 (28.1%) | 20 (15.7%) |  |
| 0.30-0.46 | 36 (8.5%) | 186 (63.1%) | 97 (76.4%) |  |
| ≥0.46 |  | 26 (8.8%) | 10 (7.9%) |  |
| **Inflammatory markers** |  |  |  |  |
| PIV | 0.24 (0.12–0.40) | 0.23 (0.11–0.40) | 0.26 (0.15–0.44) | 0.090 |
| Procalcitonin, ng/mL | 20.2 (4.6–69.2) | 21.7 (5.1–73.5) | 14.1 (3.2–54.3) | 0.051 |
| Interleukin-6, pg/mL | 193.7 (63.8–1916.5) | 283.0 (72.8–1989.0) | 139.0 (53.1–827.0) | 0.061 |
| C-reactive protein, mg/L | 156.9 (74.9–262.5) | 153.7 (74.9–272.4) | 166.6 (73.3–249.5) | 0.578 |
| **Electrolytes and Renal function** |  |  |  |  |
| Potassium, mmol/L | 4.0 (3.5–4.5) | 4.0 (3.5–4.5) | 4.0 (3.5–4.5) | 0.738 |
| Sodium, mmol/L | 137.5 (133.0–142.0) | 137.6 (133.0–141.9) | 137.2 (133.5–143.3) | 0.497 |
| Urea/Creatinine ratio | 42.1 (33.3–55.2) | 41.5 (32.9–54.7) | 44.8 (34.2–56.2) | 0.490 |
| eGFR, mL/min/1.73m² | 35.2 (22.3–59.3) | 33.7 (21.1–55.5) | 39.4 (24.4–66.6) | 0.077 |
| **Liver function and Lipids** |  |  |  |  |
| Total bilirubin, μmol/L | 17.9 (10.7–36.0) | 18.0 (10.8–35.1) | 17.4 (10.6–38.0) | 0.977 |
| Direct bilirubin, μmol/L | 10.5 (5.9–24.0) | 10.4 (6.1–24.3) | 10.5 (5.0–24.0) | 0.478 |
| ALT, U/L | 28.0 (15.0–66.8) | 28.0 (15.0–76.5) | 28.0 (16.5–58.5) | 0.819 |
| AST, U/L | 39.0 (24.0–109.8) | 40.0 (23.5–120.0) | 38.0 (25.0–90.5) | 0.833 |
| Albumin, g/L | 28.8 (25.3–33.0) | 28.8 (25.2–33.0) | 28.8 (26.1–33.4) | 0.755 |
| Total cholesterol, mmol/L | 2.6 (2.1–3.4) | 2.6 (2.1–3.4) | 2.8 (2.2–3.5) | 0.191 |
| Triglycerides, mmol/L | 1.5 (0.9–2.2) | 1.4 (0.9–2.1) | 1.6 (1.0–2.3) | 0.167 |
| **Cardiac markers** |  |  |  |  |
| Cardiac troponin, ng/L | 0.05 (0.02–0.19) | 0.06 (0.02–0.18) | 0.05 (0.02–0.22) | 0.401 |
| NT-proBNP, pg/mL | 3240.0 (1014.8–8746.5) | 3396.0 (1016.5–8829.0) | 3122.0 (984.5–8694.0) | 0.746 |
| **Coagulation profile** |  |  |  |  |
| Fibrinogen, g/L | 4.2 (3.0–5.3) | 4.3 (3.0–5.4) | 4.1 (3.1–5.3) | 0.861 |
| FDP, mg/L | 16.6 (9.1–35.9) | 16.4 (9.3–34.2) | 16.7 (8.4–38.8) | 0.920 |
| D-dimer, mg/L FEU | 3.0 (1.2–7.3) | 3.1 (1.3–7.2) | 2.8 (1.1–8.0) | 0.584 |
| INR | 1.2 (1.1–1.5) | 1.3 (1.1–1.5) | 1.2 (1.1–1.5) | 0.589 |
| PT, s | 13.8 (12.2–16.5) | 14.0 (12.3–16.3) | 13.5 (12.1–16.7) | 0.348 |
| APTT, s | 32.6 (28.4–37.4) | 32.7 (28.6–37.4) | 32.3 (28.1–37.2) | 0.497 |
| Prothrombin activity, % | 72.0 (55.9–88.0) | 71.0 (56.0–86.4) | 74.0 (55.0–90.0) | 0.408 |
| **Outcomes** |  |  |  |  |
| Renal function recovery at 28 days | 197 (46.7%) | 140 (47.5%) | 57 (44.9%) | 0.627 |
| Lactate clearance within 48h | 236 (55.9%) | 160 (54.2%) | 76 (59.8%) | 0.287 |
| RRT during hospitalization | 165 (39.1%) | 116 (39.3%) | 49 (38.6%) | 0.886 |
| Tracheal intubation | 144 (34.1%) | 104 (35.3%) | 40 (31.5%) | 0.455 |

Abbreviations: SA-AKI: Sepsis-Associated Acute Kidney Injury; E-SA-AKI: Early SA-AKI; L-SA-AKI: Late SA-AKI; BMI: Body Mass Index; SOFA: Sequential Organ Failure Assessment; APACHE II: Acute Physiology and Chronic Health Evaluation II; GCS: Glasgow Coma Scale; AKI: Acute Kidney Injury; MAP: Mean Arterial Pressure; PaO/FiO: Ratio of arterial oxygen partial pressure to fractional inspired oxygen; WBC: White Blood Cell count; PIV: Pan-Immune-Inflammation Value; eGFR: estimated Glomerular Filtration Rate; ALT: Alanine Aminotransferase; AST: Aspartate Aminotransferase; NT-proBNP: N-terminal pro-B-type Natriuretic Peptide; FDP: Fibrin Degradation Products; INR: International Normalized Ratio; PT: Prothrombin Time; APTT: Activated Partial Thromboplastin Time; RRT: Renal Replacement Therapy. Data are presented as n (%), median (IQR), or mean ± SD.

**Supplemental Table 3**. Variables associated with the 1-year mortality according to the univariate Cox regression analysis

| **Variable** | **HR (95%CI)** | **P value** |
| --- | --- | --- |
| **Stage(L-SA-AKI)** | 1.71 (1.26, 2.32) | **0.001** |
| **Age** | 1.01 (0.99, 1.02) | 0.052 |
| **Sex (Male)** | 1.51 (1.07, 2.13) | **0.019** |
| **BMI, kg/m^2^** |  |  |
| <18.5 | 2.07 (1.08, 3.97) | **0.028** |
| ≥25.0-28.0 | 0.80 (0.58, 1.11) | 0.177 |
| ≥28 | 0.88 (0.47, 1.64) | 0.694 |
| **SOFA score** | 1.04 (0.99, 1.08) | 0.106 |
| **APACHEII score** | 1.07 (1.05, 1.09) | **<0.001** |
| **GCS score** | 0.96 (0.92, 1.01) | 0.064 |
| **Comorbidities** |  |  |
| Hypertension | 1.02 (0.76, 1.36) | 0.902 |
| Diabetes | 1.13 (0.84, 1.53) | 0.408 |
| Coronary heart disease | 0.97 (0.67, 1.39) | 0.860 |
| Chronic heart dyfunction | 1.35 (0.96, 1.89) | 0.084 |
| Cerebrovascular disease | 1.08 (0.77, 1.52) | 0.660 |
| Chronic pulmonary disease | 1.08 (0.64, 1.83) | 0.775 |
| Chronic liver disease | 1.61 (0.89, 2.89) | 0.113 |
| Malignancy | 1.85 (0.87, 3.95) | 0.109 |
| Rheumatoid arthritis | 1.27 (0.63, 2.58) | 0.507 |
| Cardiac arrest | 3.45 (1.94, 6.14) | **<0.001** |
| **Number of organ failures** | 1.20 (0.95, 1.51) | 0.129 |
| **New complications** |  |  |
| Myocardial infarction | 1.15 (0.54, 2.46) | 0.711 |
| Acute heart failure | 1.12 (0.79, 1.58) | 0.520 |
| **Septic shock** | 2.94 (1.90, 4.56) | **<0.001** |
| **Site of infection** |  |  |
| Non-abdominal (lung, urinary) vs Abdominal | 1.34 (0.97, 1.86) | 0.076 |
| Other (intracranial, bloodstream, skin/soft tissue) vs Abdominal | 1.07 (0.93,1.58) | 0.735 |
| **AKI stage** |  |  |
| 2 vs 1 | 1.38 (0.84, 2.26) | 0.199 |
| 3 vs 1 | 2.47 (1.57, 3.87) | **<0.001** |
| **Physiological parameters** |  |  |
| MAP, mmHg | 0.99 (0.99, 1.01) | 0.061 |
| Heart rate, bpm | 1.01 (0.99, 1.01) | 0.064 |
| Respiratory rate, breaths/min | 1.01 (0.99, 1.03) | 0.108 |
| Temperature, °C | 1.03 (0.88, 1.20) | 0.752 |
| **First 24-hour data** |  |  |
| Urine output, ml | 0.86 (0.80, 0.92) | **<0.001** |
| Fluid balance(>0 mL) | 1.09 (0.77, 1.53) | 0.624 |
| **Vasoactive agent duration, days** | 1.05 (1.00, 1.11) | 0.069 |
| **Laboratory parameters** |  |  |
| Calculated oxygenation index (PaO₂/FiO₂) (≥200) | 0.62 (0.46, 0.82) | **0.001** |
| PaCO2 | 1.02 (1.01, 1.03) | **0.004** |
| PH(≥7.35) | 0.55 (0.41, 0.73) | **<0.001** |
| Hematocrit |  |  |
| 0.30-0.46 vs <0.30 | 1.30 (0.95, 1.79) | 0.104 |
| ≥0.46 vs <0.30 | 0.83 (0.48, 1.45) | 0.520 |
| HCO₃⁻ | 0.99 (0.97, 1.02) | 0.711 |
| ln(Lactate) | 1.20 (0.97, 1.49) | 0.098 |
| Base excess | 0.99 (0.96, 1.01) | 0.279 |
| WBC | 0.87 (0.65, 1.18) | 0.372 |
| ln(Platelet) | 0.98 (0.82, 1.18) | 0.858 |
| NE | 0.99 (0.97, 1.01) | 0.070 |
| **Inflammatory markers** |  |  |
| ln(PIV) | 0.97 (0.82, 1.13) | 0.678 |
| ln(Procalcitonin) | 0.94 (0.87, 1.02) | 0.136 |
| ln(Interleukin-6) | 1.07 (0.99, 1.14) | 0.058 |
| ln(C-reactive protein) | 0.99 (0.86, 1.13) | 0.839 |
| **Electrolytes and Renal function** |  |  |
| ln(Potassium) | 2.38 (1.13, 5.00) | **0.022** |
| ln(Sodium) | 1.14 (0.35, 3.74) | 0.827 |
| ln(Urea/Creatinine ratio) | 1.28 (0.98, 1.67) | 0.071 |
| ln(eGFR) | 1.24 (0.99, 1.56) | 0.054 |
| **Liver function and Lipids** |  |  |
| ln(Total bilirubin) | 1.06 (0.91, 1.25) | 0.444 |
| ln(Direct bilirubin) | 1.10 (0.96, 1.26) | 0.185 |
| ln(ALT) | 0.94 (0.84, 1.04) | 0.223 |
| ln(AST) | 0.99 (0.88, 1.11) | 0.874 |
| ln(Albumin) | 0.25 (0.13, 0.46) | **<0.001** |
| ln(Total cholesterol) | 0.79 (0.56, 1.12) | 0.189 |
| ln(Triglycerides) | 1.09 (0.92, 1.29) | 0.330 |
| **Cardiac markers** |  |  |
| ln(Cardiac troponin) | 0.93 (0.85, 1.02) | 0.106 |
| ln(NT-proBNP) | 0.96 (0.87, 1.05) | 0.370 |
| **Coagulation profile** |  |  |
| ln(Fibrinogen) | 0.61 (0.47, 0.80) | **<0.001** |
| ln(FDP) | 0.93 (0.81, 1.08) | 0.347 |
| ln(D-dimer) | 0.97 (0.86, 1.09) | 0.603 |
| ln(INR) | 1.12 (0.71, 1.77) | 0.615 |
| ln(PT) | 1.34 (0.82, 2.20) | 0.240 |
| ln(APTT) | 1.18 (0.69, 2.03) | 0.541 |
| ln(Prothrombin activity) | 0.86 (0.58, 1.26) | 0.442 |
| **Outcomes** |  |  |
| Renal function recovery at 28 days | 0.18 (0.13, 0.26) | **<0.001** |
| Lactate clearance within 48h | 0.27 (0.20, 0.37) | **<0.001** |
| RRT during hospitalization | 2.09 (1.56, 2.80) | **<0.001** |
| Tracheal intubation | 1.30 (0.97, 1.76) | 0.080 |

Abbreviations: L-SA-AKI: Late SA-AKI; BMI: Body Mass Index; SOFA: Sequential Organ Failure Assessment; APACHE II: Acute Physiology and Chronic Health Evaluation II; GCS: Glasgow Coma Scale; AKI: Acute Kidney Injury; MAP: Mean Arterial Pressure; PaO/FiO: Ratio of arterial oxygen partial pressure to fractional inspired oxygen; WBC: White Blood Cell count; PIV:Pan-Immune-Inflammation Value; eGFR: estimated Glomerular Filtration Rate; ALT: Alanine Aminotransferase; AST: Aspartate Aminotransferase; NT-proBNP: N-terminal pro-B

type Natriuretic Peptide; FDP: Fibrin Degradation Products; INR: International Normalized Ratio; PT: Prothrombin Time; APTT: Activated Partial Thromboplastin Time; RRT: Renal Replacement Therapy.

**Supplemental Figure 1.** The number of survivors and deaths of days from sepsis onset

**Supplemental Figure 2.** Lasso analysis based on the factors with univariate Cox regression analysis
